# Supplementary material for: A regulator of amino acid catabolism controls Acinetobacter baumannii gut colonization
Source: Nat Commun. 2026 Aug 14;17:9773. doi: 10.1038/s41467-026-75861-5 (PMC13572408; doi:10.1038/s41467-026-75861-5)

Supplementary Information

A regulator of amino acid catabolism controls *Acinetobacter baumannii* gut colonization

John H. Geary[1,&], Xiaomei Ren[1,&], Dziedzom A. Bansah[1], Ngoc T.T. Pham[2], Iván C. Acosta[1], Jim M. Camilleri[1], Bryan M. Kigongo[1], Jonathan D. Winkelman[3], Francis Alonzo III[1], Matthew T. Henke[2], Lauren D. Palmer[1,*]

[1]Department of Microbiology & Immunology, University of Illinois Chicago, Chicago, IL, USA
[2]Department of Pharmaceutical Sciences, University of Illinois Chicago, Chicago, IL, USA
[3]Trestle, LLC, Milwaukee, WI, USA
[*]Correspondence: ldpalmer@uic.edu
[&]Contributed equally

A

```
                                 +                    +     ++  + + +            +   +    +
AstR A. baylyi                 MNDTDSQILGLLRDNARMSVTDLAAKVKVSRATIQKRIEYMENQGIITGYTVRIKPNSEQ   60
AstR A. colistiniresistens     MNNIDHIILGLLKDNARMSITDLAAKTRVSRATVQKRIEYMESTGVITGYTVRFRPNTEK   60
AstR A. baumannii              MDNIDQIILGLLKDNARISVTELAEKVHVSRATIKKRMEYLESSGIITGYTVRFKPNAER   60
                               *:: *  *****:****:*:*:** *.:*****::**:**:*. *:*******::**:*:
                                                                     +
                                   +           +       +        ## # ##     +     +     #
AstR A. baylyi                 NMIRAWMNIMVEGNKASAVIRELRIDPAVHTLHTTNGKWDLLVELKSENLASFDKILERI   120
AstR A. colistiniresistens     NIIRAWMNIMVEGTRAQAVIRELRLESAVERLHTTNGKWDLLVELQSDSLENFDKALERI   120
AstR A. baumannii              NVIRAWMSIMVEGTKAQSVIKELRLESAVECLHKTNGKWDILVELRSDTLENFDKVLERI   120
                               *:*****.*****.:*.:**:***:: **. **.******:****:*:.* .*** ****

                               #       ###
AstR A. baylyi                 RTINGIYNTETSILLSTYKA  140
AstR A. colistiniresistens     RNISGIYNSETSILLSTYKT  140
AstR A. baumannii              RNISGIYNSETSILLASHKT  140
                               *.*.****:******:::*:
```

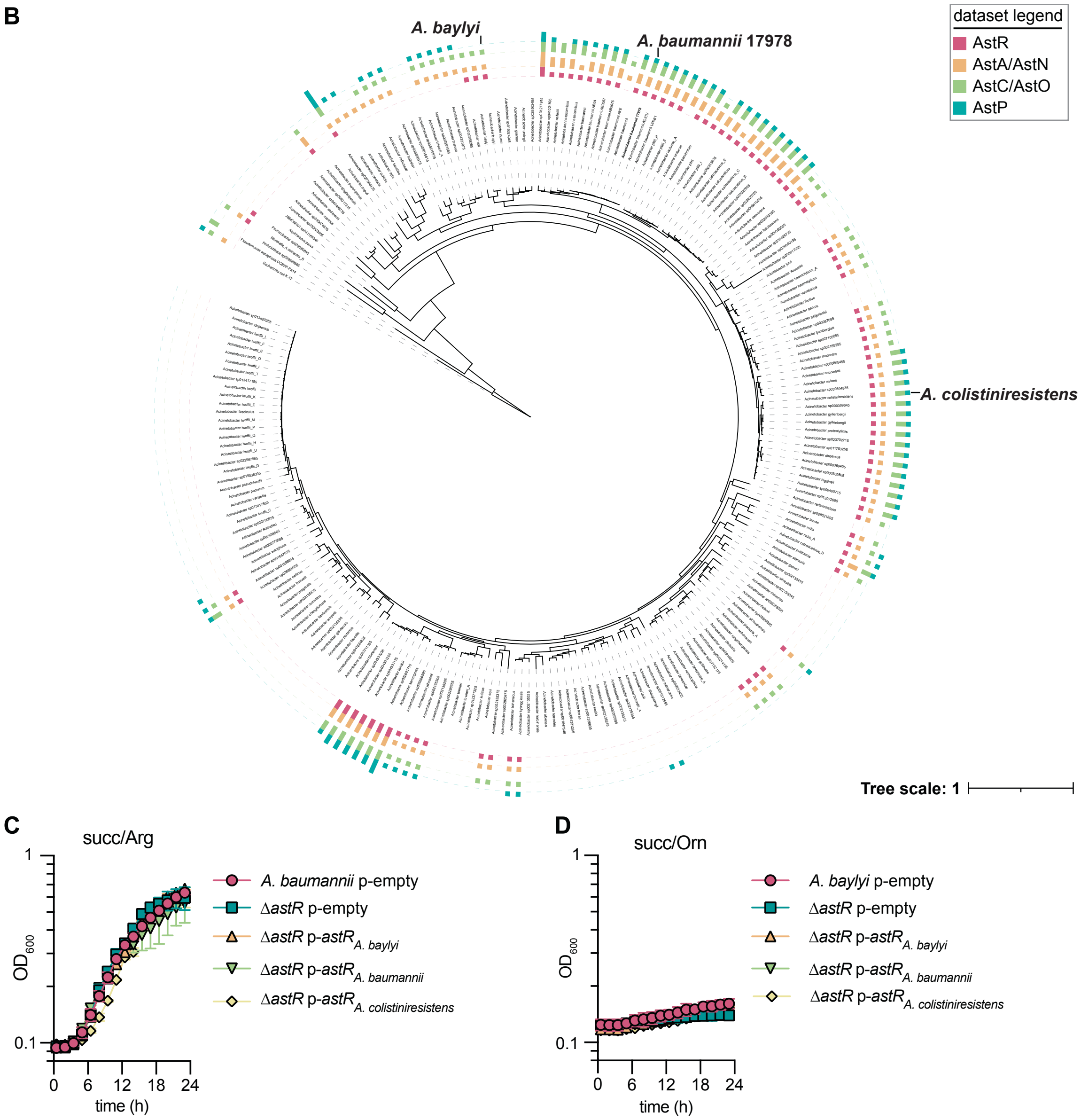

Figure S1: Additional data of *Acinetobacter* AstR.
(**A**) Multiple sequence alignment of AstR proteins encoded by *A. baylyi* ATCC ADP1, *A. colistiniresistens* NIPH 2036, and *A. baumannii* ATCC 17978[1]. Above the sequence, important residues from *E. coli* AsnC are marked with # for ligand binding and + for DNA binding[2].
(**B**) AstR and AstNOP and their corresponding copy numbers were mapped to an *Acinetobacter* species phylogenetic tree generated from 221 published *Acinetobacter* genomes (see Supplementary Data 1).

(C-D) *A. baumannii* ATCC 17978VU or *A. baylyi* ATCC ADP1 wildtype and Δ*astR* strains with empty or *astR* complementation introduced in the pWH1266 plasmid. The pWH1266 plasmid used endogenous the *astR* promoter from the host strain. Strains were grown in nitrogen-free M9 minimal media with succinate at 16.5 mM and arginine or ornithine at 18.6 mM, as indicated (n = 3 bacterial cultures; mean ± SD, experiments were performed at least twice with similar results).

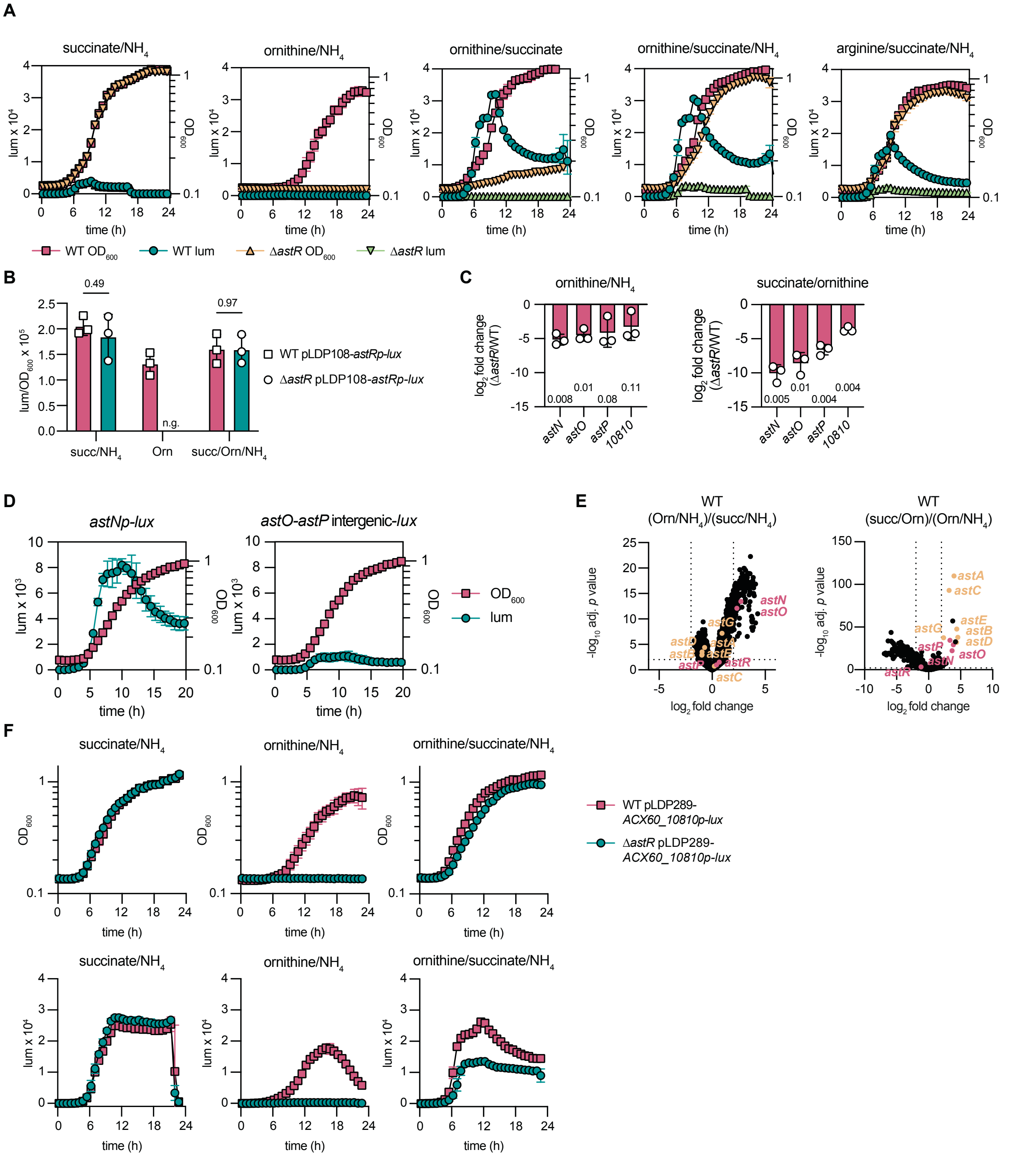

Figure S2: Additional data of *A. baumannii* wildtype and Δ*astR* strains with luciferase reporters of *astNOP*, *astR*, and *ACX60_10810* promoter activity.

(A) *A. baumannii* ATCC 17978 wildtype (WT) and Δ*astR* strains containing an *astN* promoter (*astNp*) driving luciferase expression from the *lux* operon were grown in nitrogen-free M9 minimal media minimal media with succinate (succ), ornithine (Orn), and arginine at 16.5 mM and NH_4Cl at 18.5 mM, as indicated (n = 3 bacterial cultures; mean ± SD, experiments were performed at least twice with similar results).

(B) *A. baumannii* ATCC 17978 wildtype (WT) and Δ*astR* strains containing an *astN* promoter (*astNp*) driving luciferase expression from the *lux* operon were grown in nitrogen-free M9 minimal media minimal media with succinate (succ), ornithine (Orn), and arginine at 16.5 mM and NH_4Cl at 18.5 mM, as indicated. Luminescence

was normalized to optical density at 600 nm (OD_{600}) at 0.25. n.g. indicates no growth in that condition (n = 3 bacterial cultures; mean ± SD; *p* by multiple unpaired *t*-tests; experiments were performed at least twice with similar results).
(C) qRT-PCR of genes significantly changed in RNA seq. Cycle threshold difference (-ΔCT) compared to *16s* (n = 3 bacterial cultures, *p* by Wilcoxon signed ranked test).
(D) *A. baumannii* ATCC 17978 wildtype containing an *astNp* as a positive control or the *astO-astP* intergenic region driving luciferase expression from the *lux* operon were grown in nitrogen-free M9 minimal media minimal media with succinate (16.5 mM) and ornithine (18.6 mM) (n = 3 bacterial cultures; mean ± SD, experiments were performed at least twice with similar results).
(E) RNA-seq of *A. baumannii* ATCC 17978 wildtype (WT) incubated for 1 h in M9 minimal medium with succinate (succ, 16.5 mM) and ornithine (Orn, 18.6 mM), Orn (16.5 mM) and NH_4Cl (18.6 mM), or succ (16.5 mM) and NH_4Cl (18.6 mM) as the sole carbon and nitrogen sources, as indicated. The *ast* genes are colored and labeled. Each dot is one gene (n = 3 bacterial cultures; adj. *p* value and fold-change were determined by DESeq2; dotted lines shown at significance cut-offs |log2 fold change|=2 and adj. *p* value = 0.05).
(F) *A. baumannii* ATCC 17978 wildtype (WT) and Δ*astR* strains containing an *ACX60_10810* promoter (*ACX60_10810p*) driving luciferase expression from the *lux* operon were grown in nitrogen-free M9 minimal media minimal media with succinate (succ) and ornithine (Orn) at 16.5 mM and NH_4Cl at 18.6 mM, as indicated (n = 3 bacterial cultures; mean ± SD, experiments were performed at least twice with similar results).
OD_{600}, optical density at 600 nm; WT, wildtype; *astNp, astNOP* operon promoter; lum, luminescence; *astRp*, *astR* promoter; succ, succinate; Orn, ornithine; *10810p*, *ACX60_10810* promoter.

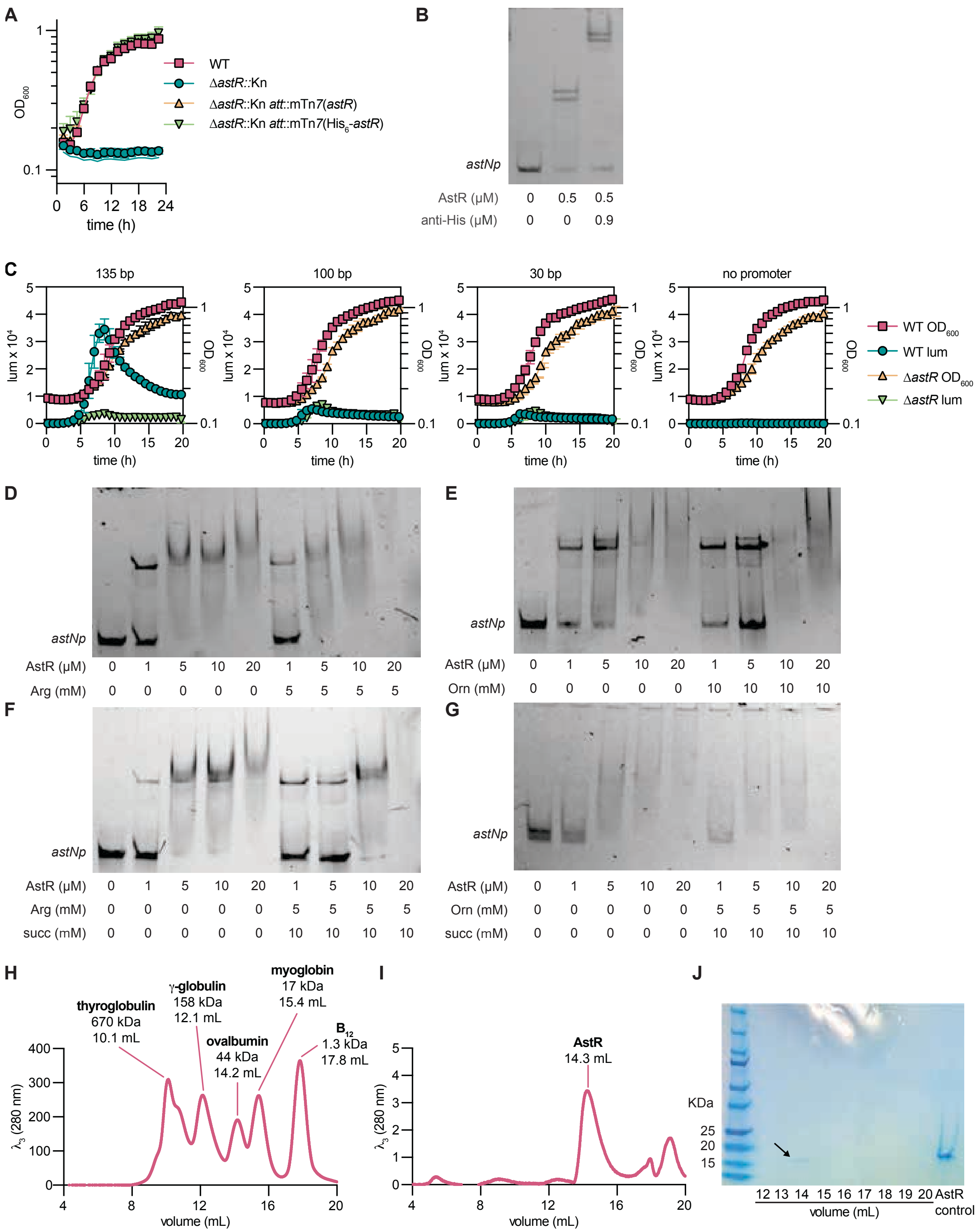

Figure S3: Additional data for His6-AstR function and oligomerization state.
(A) *A. baumannii* ATCC 17978 wildtype (WT) and Δ*astR* strains containing mTn*7* with wildtype or His$_6$-AstR were grown in nitrogen-free M9 minimal medium with ornithine (16.5 mM) as the sole carbon and nitrogen source (n = 3, mean ± SD, experiments were performed at least twice with similar results).
(B) His$_6$-AstR, Cy5.5-labeled *astNp*, and anti-His antibody were incubated together at the indicated concentrations for electrophoretic mobility shift assays (EMSA). The experiment was performed once.
(C) The 135 bp, 100 bp, and 30 bp intergenic regions upstream of *astN* were cloned upstream of a *lux* luciferase reporter or with a no promoter control. WT and Δ*astR* strains with the *lux* reporters were incubated in nitrogen-

free M9 minimal medium with ornithine at 16.5 mM, succinate at 16.5 mM, and NH_4Cl at 18.6 mM. (n = 3; mean ± SD, experiments were performed at least twice with similar results).
(D-G) His_6-AstR, Cy5.5-labeled *astNp*, and indicated metabolites were incubated together at the indicated concentrations for EMSA. Experiments were performed at least the following number of times with similar results: D, 3; E, 2; F, 2; G, 3.
(H-I) Size exclusion chromatography of standards and His_6-AstR. The experiment was performed twice with similar results.
(J) Polyacrylamide gel of size exclusion fractions stained with SimplyBlue confirm the peak at 14 mL is likely AstR.
OD_{600}, optical density at 600 nm; mTn*7*, mini Tn*7*; *astNp, astNOP* operon promoter; lum, luminescence; Orn, ornithine.

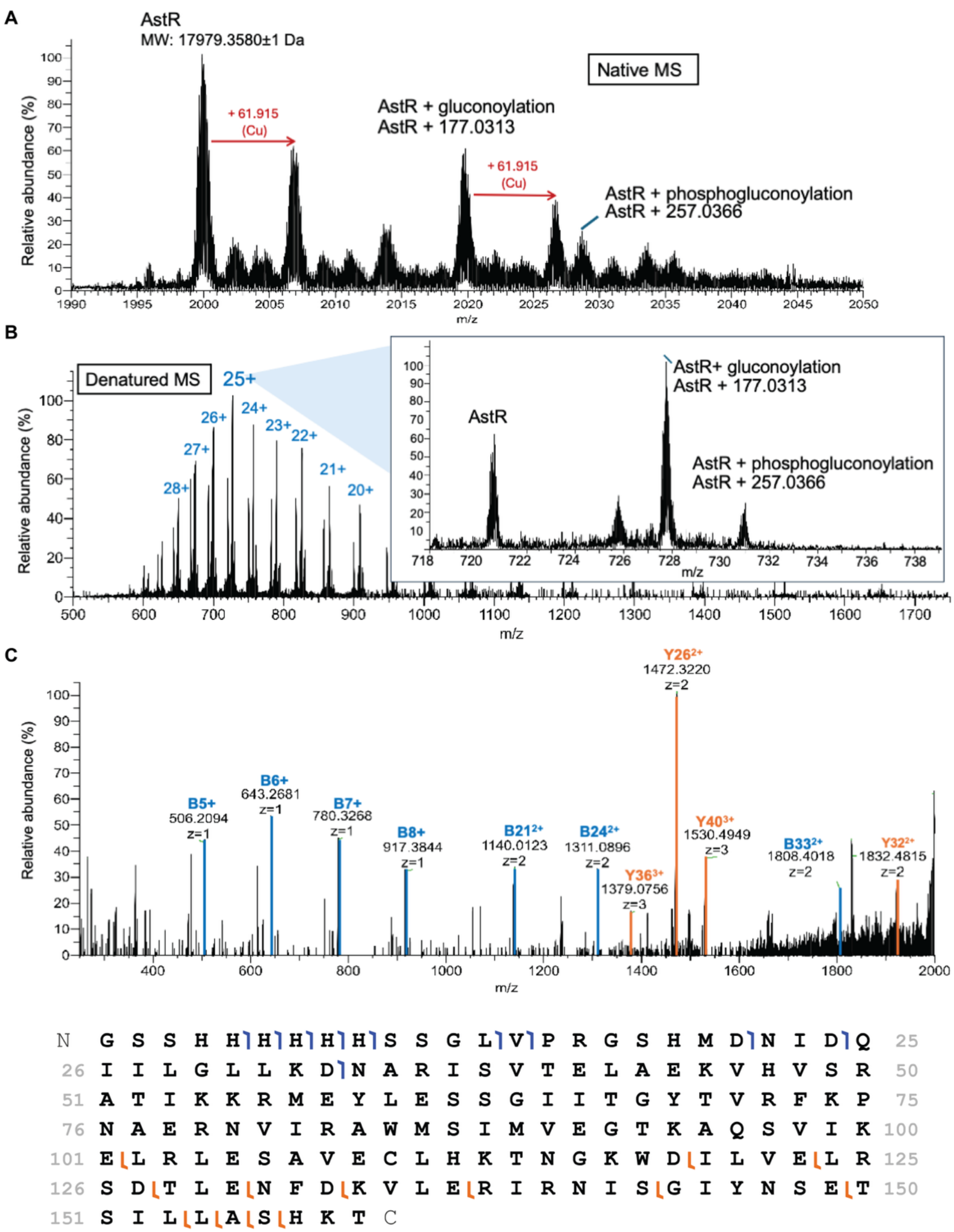

Figure S4: Purified His$_6$-AstR did not appear to bind any ligand as determined by native and denatured mass spectrometry.
(A) Native mass spectrometry (MS) showed a complex spectrum indicating many distinct states of AstR. AstR appeared with the removal of N-formylmethionine. It was futher modified with α-N-gluconoylation (+177.0313±1. Da) and α-N-6-phosphogluconoylation (+257.0366±1Da), supported by Geoghegan *et al.*, 1998[3]. Both AstR and modified AstR potentially bound to copper.

(B) Denatured LC-MS showed similar features of modified AstR.
(C) Tandem mass spectrum (MS2) of AstR with annotations of a few highly abundant b and y ions, which were detected and mapped by ProSight Lite[4]. Data representative of analysis from two independently purified protein preparations.
MS, mass spectrometry; Da, daltons; *m/z*, mass-to-charge ratio.

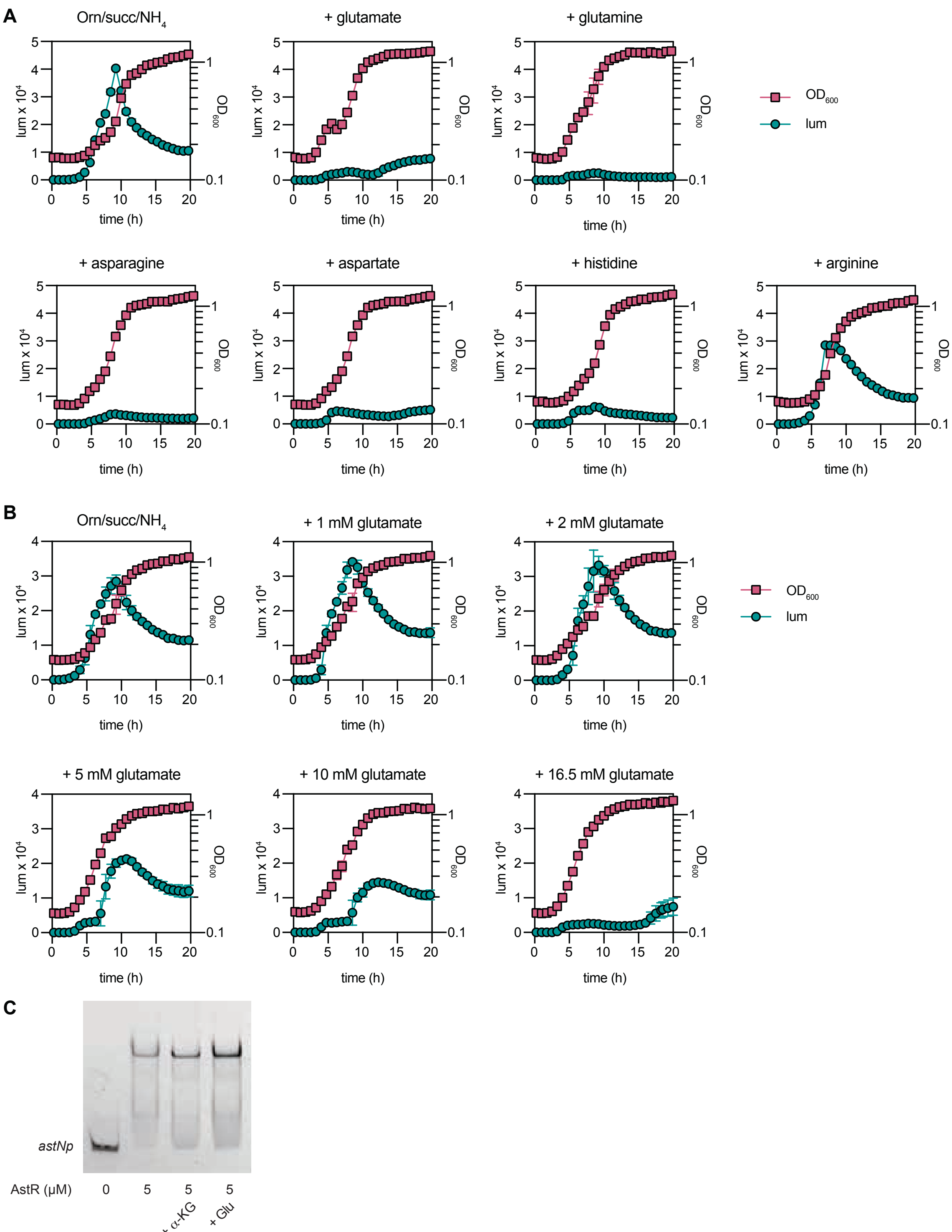

Figure S5: Growth, luminescence, and EMSA in the presence of preferred amino acid carbon sources such as glutamate.

(A-B) *A. baumannii* ATCC 17978 wildtype containing an *astN* promoter (*astNp*) driving luciferase expression from the *lux* operon was grown in nitrogen-free M9 minimal media minimal media with succinate (succ) and ornithine (Orn) at 16.5 mM and NH_4Cl at 18.5 mM. In (A), 16.5 mM additional amino acids were added as indicated. In (B), glutamate was added at the indicated concentrations. Luminescence and optical density at 600 nm (OD_{600}) are shown (n = 3 bacterial cultures, mean ± SD, *p* by one-way ANOVA with Dunnett's multiple comparisons to no additional amino acids; experiments were performed at least twice with similar results)

(C) Cy5.5-labeled *astNp* DNA was incubated with His_6-AstR at the indicated concentrations and α-ketoglutarate or glutamate at 5 mM for EMSA. Experiment was performed at least twice with similar results.
OD_{600}, optical density at 600 nm; lum, luminescence; Orn, ornithine; α-KG, α-ketoglutarate.

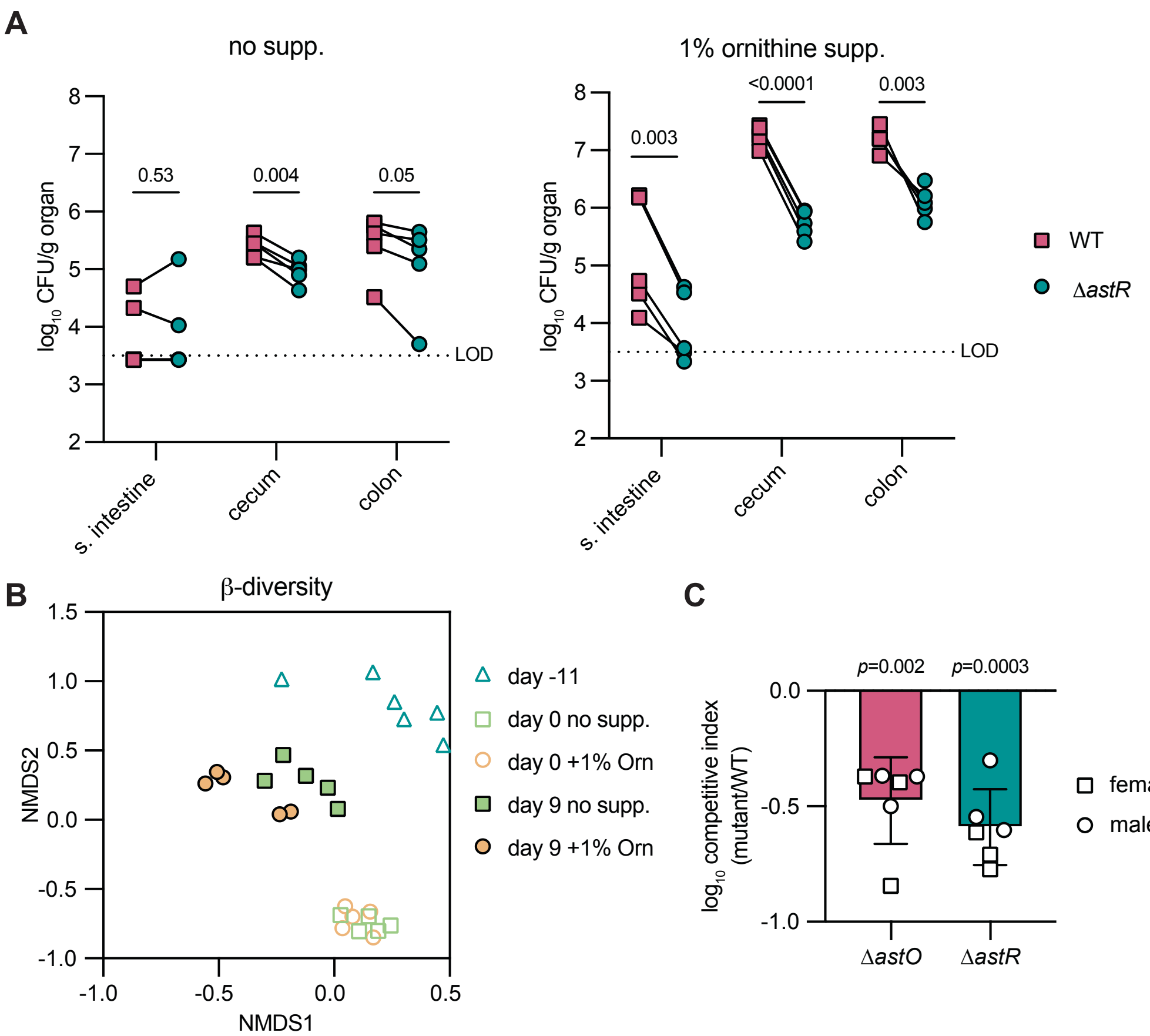

Figure S6: AstR is required for persistent gut colonization.
(A) Mice (from Figure 5A) were euthanized at 10 days post infection and *A. baumannii* CFU were enumerated from the small intestine, cecum, and colon (n = 5 mice, *p* by multiple paired t-tests).
(B) β-diversity NMDS plot of 16S rRNA gene profiling at -11, 0 and 9 DPI in the feces of post-abx female Swiss Webster mice inoculated with *A. baumannii* 17978 WT and Δ*astR* and shown in Figure 5A (n = 6 mice from day -11, n = 5 mice from day 0 and day 9).
(C) Competitive index of *A. baumannii* ATCC 17978 wildtype (WT) compared to Δ*astO* and Δ*astR* for growth in fecal slurries from conventional female and male mice.
n = 6 (fecal samples from n = 3 female mice and n = 3 male mice; mean ± SD; *p* by one-sample *t* test).
Supp., supplementation; CFU, colony forming unit; s. intestine, small intestine; NMDS, non-metric multidimensional scaling; Orn, ornithine.

SUPPLEMENTARY METHODS

Size exclusion chromatography

Purified His_6-AstR was applied to a BioRad ENrich size exclusion chromatography (SEC) 650 10 × 300 mm column connected to a BioRad NGC medium pressure chromatography system and the BioRad ChromLab software was used for analysis. The column is stored in 20% ethanol while not being used, and then washed with water before every use. The column was equilibrated with 2.5 column volumes of the SEC buffer (50 mM Tris-HCl, 300 mM NaCl pH=8.0). Following equilibration, 200 µL of the sample was injected into the valve. Elution fractions were collected in 1-mL volumes from 4 mL to 26 mL. Fractions 9-17 (the fractions that exhibited peaks and those in between in ChromLab) were separated by on sodium dodecasulfate polyacrylamide gel electrophoresis (SDS-PAGE) and stained using SimplyBlue SafeStain (ThermoFisher). To estimate the size of the AstR oligomer, gel filtration standards containing a lyophilized mix of thyroglobulin, bovine γ-globulin, chicken ovalbumin, equine myoglobin, and vit B_{12} (Bio-Rad) was passed through the column and their peaks were analyzed in ChromLab.

LC-MS, Native mass spectrometry acquisition and analysis

For native mass spectrometry (MS), purified His_6-AstR was exchanged into 50 mM ammonium acetate buffer, pH 7, using a PD-10 column (Cytiva). The sample was sterile filtered using 0.2 µm hydrophilic polyvinylidene fluoride (PVDF) syringe filters to remove large contamination. The system was first optimized with 0.1 mg/mL lysozyme solution. Ion spray parameters were set at 3000 V for spray voltage, 20 for sheath gas, 5 for aux gas, and 0 for sweep gas. About 2 µg was injected to the Orbitrap Exploris 120 (Thermo Fisher Scientific) with the flowrate of 10-20 µL/min through a low-flow metal needle. To acquire MS2 data for a specific *m/z* value, injection time was manually increased from 100 to 1000 ms to accumulate more ions. At the same time, the mass range was adjusted to collect ions of interest, decreased high-energy collisional dissociation (hcd) to 1 while collecting ions, and increased the number of microscans to 10 to verify the purity of ions in the HCD cell. Then, the HCD energy was gradually increased up to 35 for fragmentation.

For denatured analysis, the sample of His_6-AstR in 50 mM ammonium acetate buffer was injected into Vanquish HPLC connected to Obitrap Exploris 120 MS (Thermo Fisher Scientific). The protein was loaded on 250 mm monolith ProSwift™ RP-4H LC column with a flow rate of 0.150 µL/min. The mobile phase started with 95% water (with 0.1% formic acid) and gradually increased to 100% acetonitrile (with 0.1% formic acid). Data acquisition in LC-MS system was done by Xcalibur (Thermo Fisher Scientific). The spectra for both Native MS and LC-MS were averaged and analyzed by FreeStyle (Thermo Fisher Scientific).

SUPPLEMENTARY REFERENCES

1. Madeira, F. *et al.* The EMBL-EBI Job Dispatcher sequence analysis tools framework in 2024. *Nucleic Acids Research* **52**, W521–W525 (2024).
2. Thaw, P. *et al.* Structural insight into gene transcriptional regulation and effector binding by the Lrp/AsnC family. *Nucleic Acids Res* **34**, 1439–1449 (2006).
3. Geoghegan, K. F. *et al.* Spontaneous alpha-*N*-6-phosphogluconoylation of a 'His tag' in *Escherichia coli*: the cause of extra mass of 258 or 178 Da in fusion proteins. *Anal Biochem* **267**, 169–184 (1999).
4. Fellers, R. T. *et al.* ProSight Lite: graphical software to analyze top-down mass spectrometry data. *Proteomics* **15**, 1235–1238 (2015).

SUPPLEMENTARY UNCROPPED GELS

Figure S3B

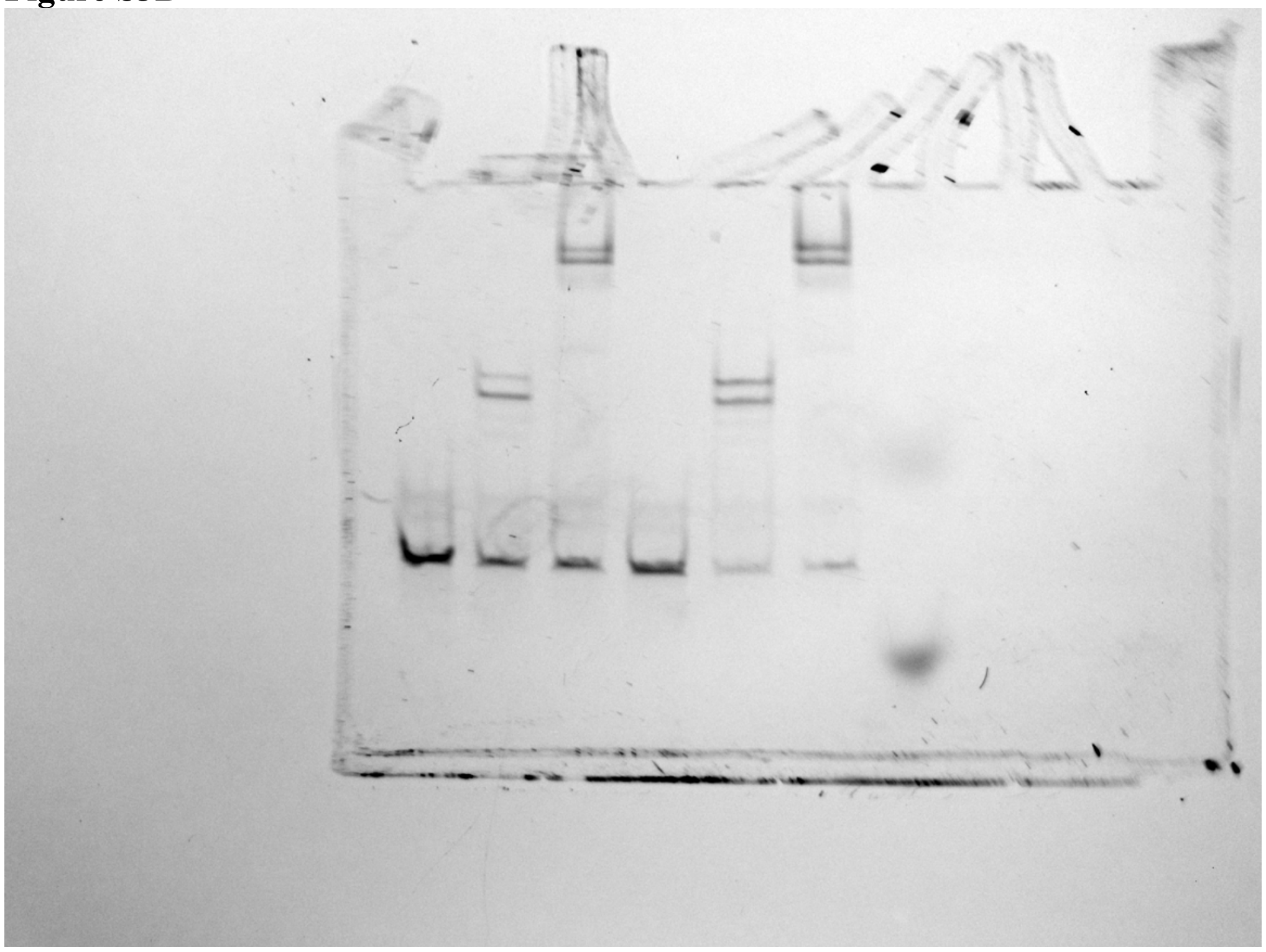

Figure S3D

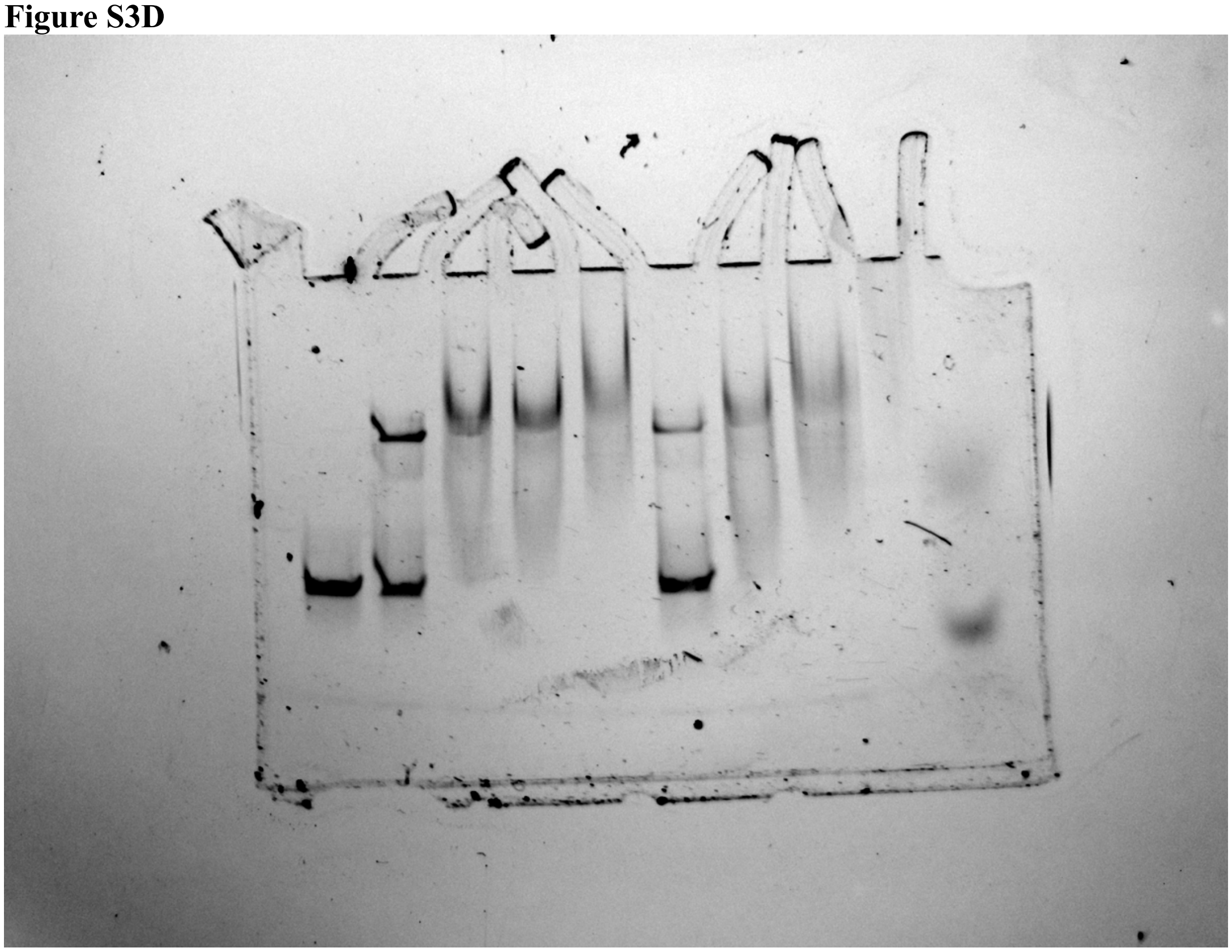

Figure S3E

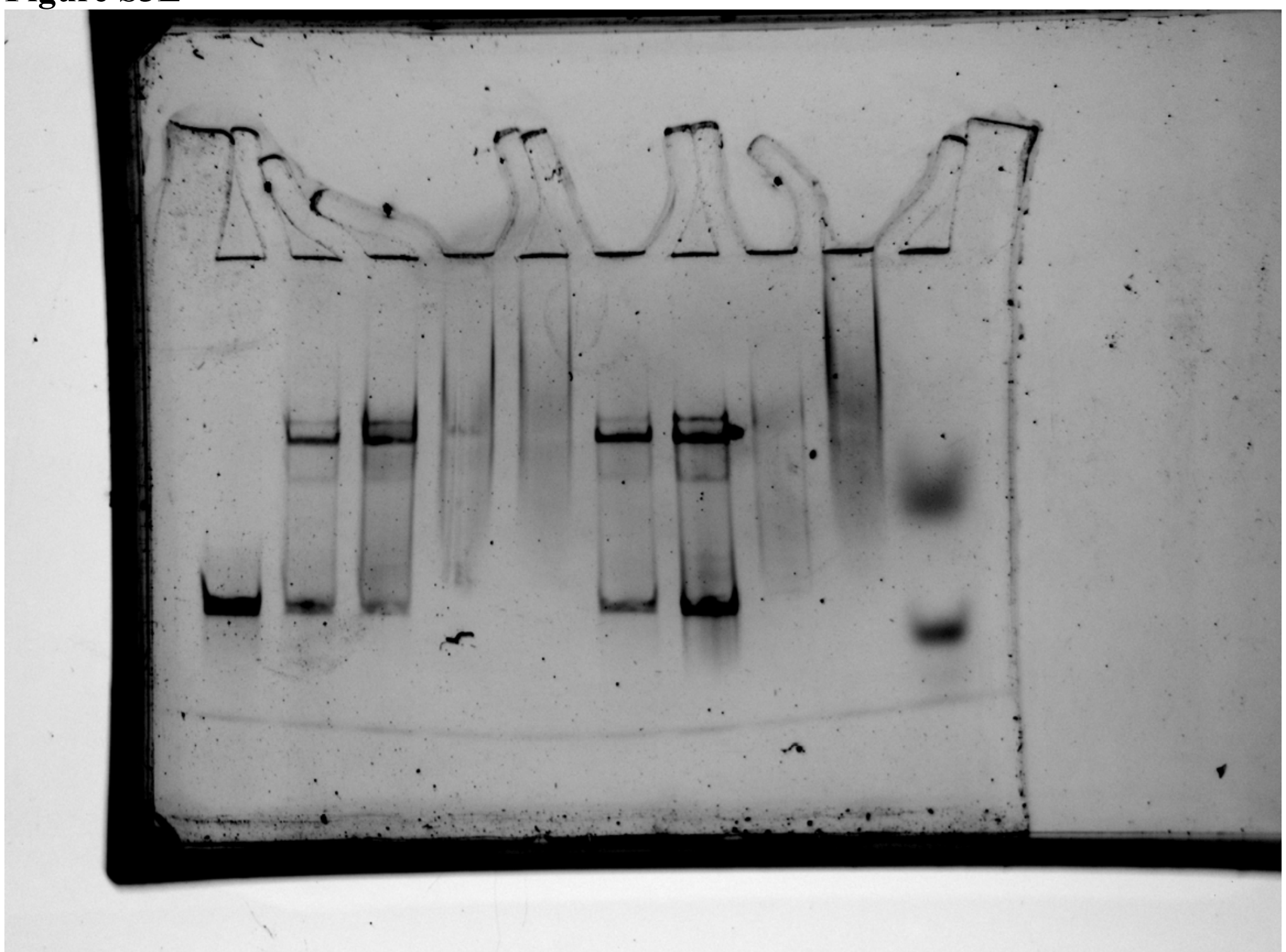

Figure S3F

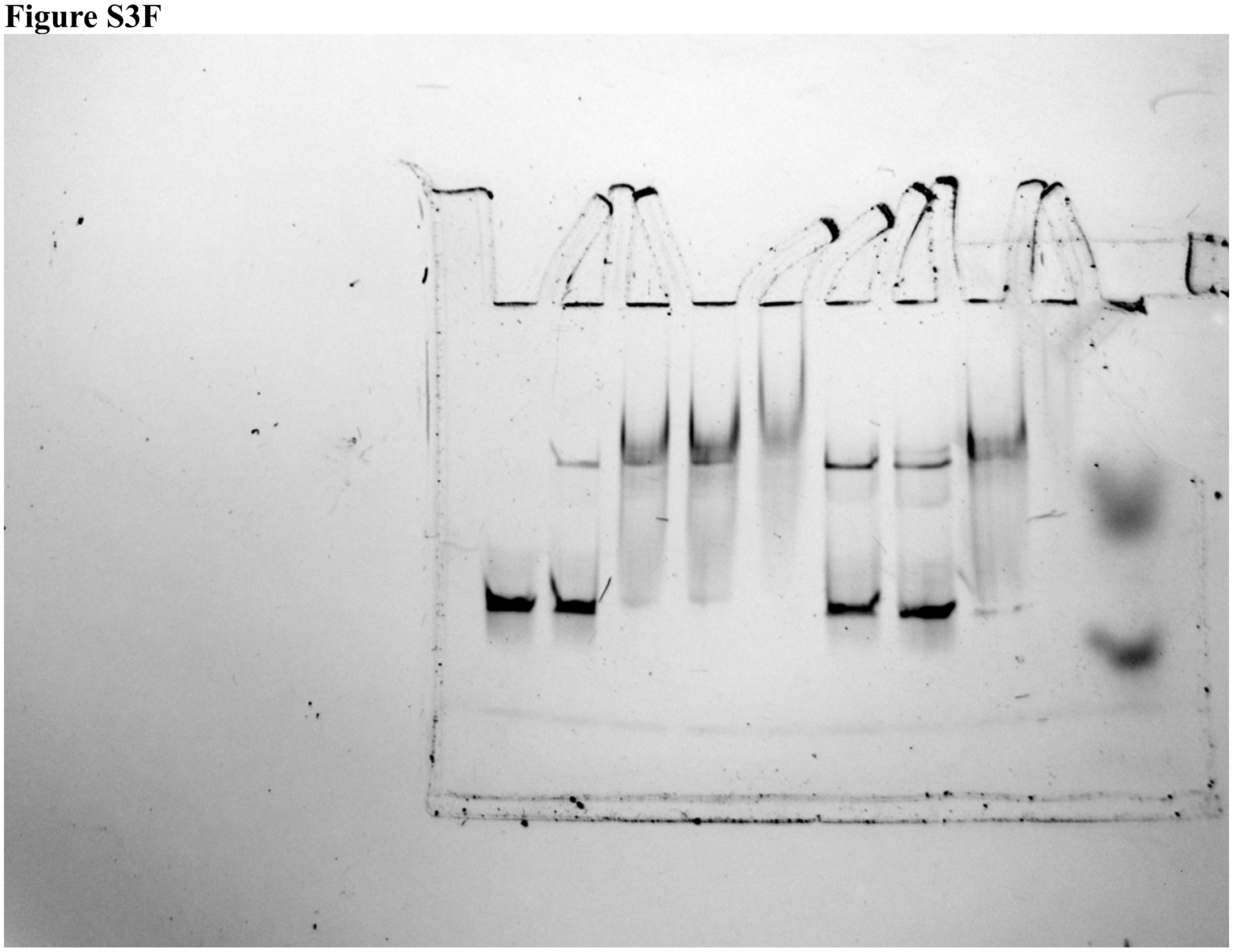

Figure S3G

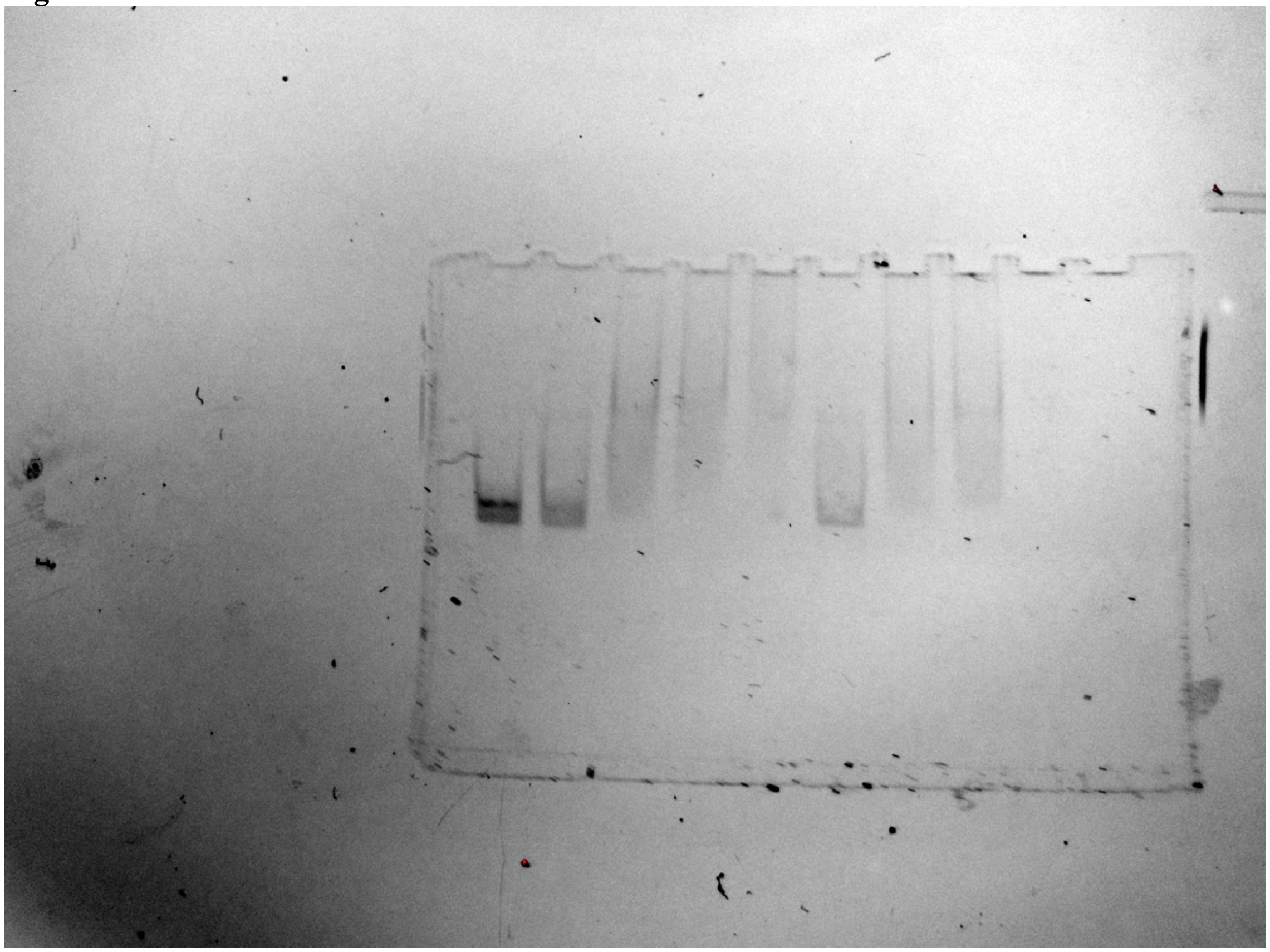

Figure S3J

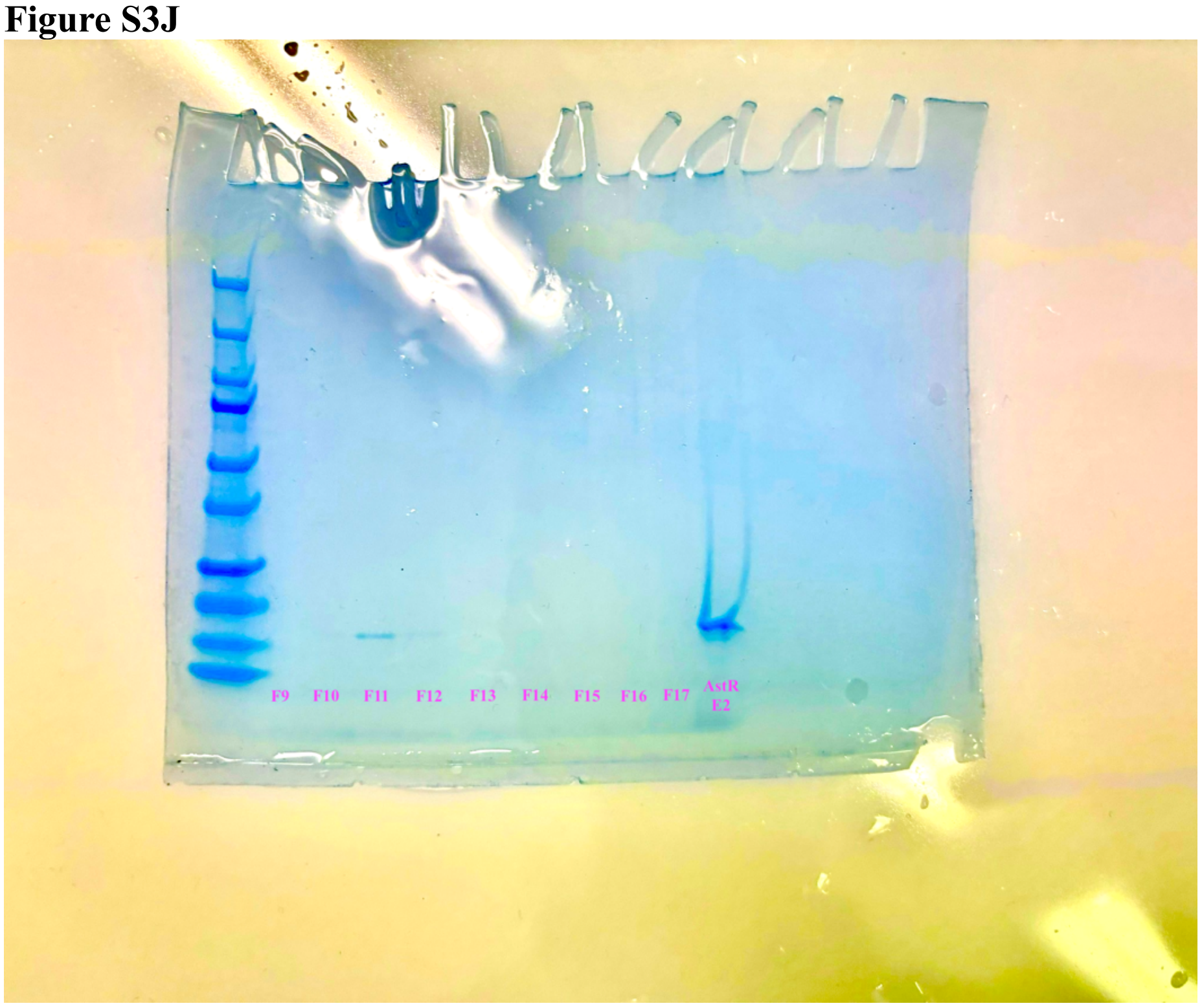

Figure S5C

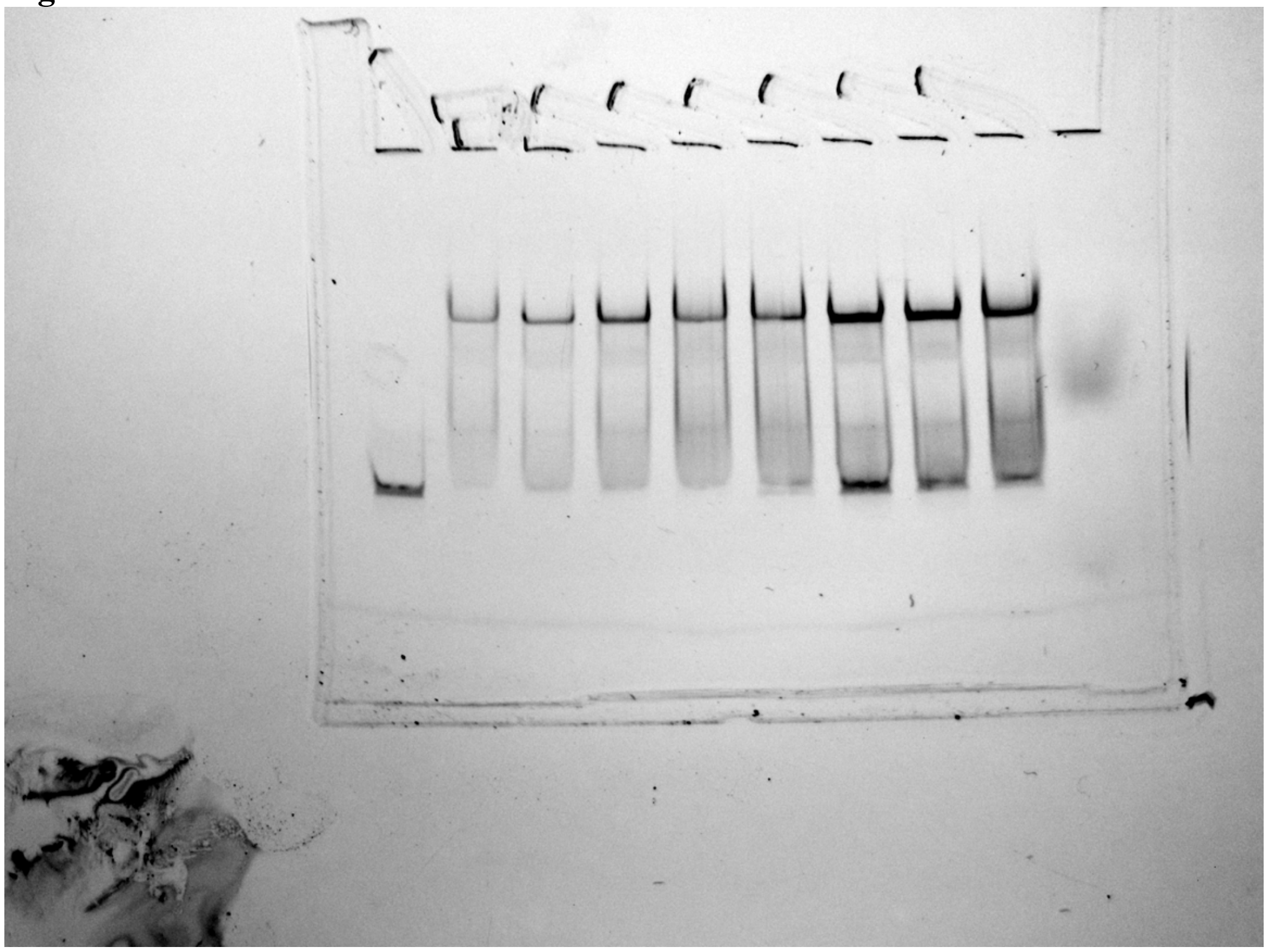

Supplement: Supplementary file 1 [file 41467_2026_75861_MOESM1_ESM.pdf]
