# Supplementary material for: A regulator of amino acid catabolism controls Acinetobacter baumannii gut colonization
Source: Nat Commun. 2026 Aug 14;17:9773. doi: 10.1038/s41467-026-75861-5 (PMC13572408; doi:10.1038/s41467-026-75861-5)
Supplement: Supplementary file 2 [file 41467_2026_75861_MOESM2_ESM.pdf]

Title: Supplementary Data 1
Description: AstR and AstNOP and their corresponding copy numbers were mapped to an *Acinetobacter* species phylogenetic tree generated from 221 published *Acinetobacter* genomes. One representative genome per species cluster was selected from the Genome Taxonomy Database (GTDB) with additional A. baumannii strains of interest. Encoding AstR, AstN, AstO, and AstP was inferred with Orthofinder.

Title: Supplementary Data 2
Description: Expression analysis of RNA-seq of WT and Δ*astR A. baumannii* strains after 1 h exposure to nitrogen-free M9 minimal medium with carbon sources added 16.5 mM and nitrogen sources to 18.6 mM as indicated (n = 3; adj. *p* value and fold-change determined by DESeq2 which uses the two-sided Wald test with Benjamini and Hochberg multiple comparisons).

Title: Supplementary Data 3
Description: Strains used in this study.

Title: Supplementary Data 4
Description: Oligonucleotides used in this study.
