# Supplementary material for: A regulator of amino acid catabolism controls Acinetobacter baumannii gut colonization
Source: Nat Commun. 2026 Aug 14;17:9773. doi: 10.1038/s41467-026-75861-5 (PMC13572408; doi:10.1038/s41467-026-75861-5)
Supplement: Supplementary file 3 [file 41467_2026_75861_MOESM7_ESM.pdf]

nature portfolio

Corresponding author(s): Lauren D. Palmer

Last updated by author(s): Jun 15, 2026

# Reporting Summary

Nature Portfolio wishes to improve the reproducibility of the work that we publish. This form provides structure for consistency and transparency in reporting. For further information on Nature Portfolio policies, see our Editorial Policies and the Editorial Policy Checklist.

## Statistics

For all statistical analyses, confirm that the following items are present in the figure legend, table legend, main text, or Methods section.

| n/a | Confirmed | |
|---|---|---|
| ☐ | ☒ | The exact sample size ($n$) for each experimental group/condition, given as a discrete number and unit of measurement |
| ☐ | ☒ | A statement on whether measurements were taken from distinct samples or whether the same sample was measured repeatedly |
| ☐ | ☒ | The statistical test(s) used AND whether they are one- or two-sided<br>*Only common tests should be described solely by name; describe more complex techniques in the Methods section.* |
| ☒ | ☐ | A description of all covariates tested |
| ☐ | ☒ | A description of any assumptions or corrections, such as tests of normality and adjustment for multiple comparisons |
| ☐ | ☒ | A full description of the statistical parameters including central tendency (e.g. means) or other basic estimates (e.g. regression coefficient) AND variation (e.g. standard deviation) or associated estimates of uncertainty (e.g. confidence intervals) |
| ☐ | ☒ | For null hypothesis testing, the test statistic (e.g. $F$, $t$, $r$) with confidence intervals, effect sizes, degrees of freedom and $P$ value noted<br>*Give P values as exact values whenever suitable.* |
| ☒ | ☐ | For Bayesian analysis, information on the choice of priors and Markov chain Monte Carlo settings |
| ☒ | ☐ | For hierarchical and complex designs, identification of the appropriate level for tests and full reporting of outcomes |
| ☒ | ☐ | Estimates of effect sizes (e.g. Cohen's $d$, Pearson's $r$), indicating how they were calculated |

*Our web collection on statistics for biologists contains articles on many of the points above.*

## Software and code

Policy information about availability of computer code

| | |
|---|---|
| Data collection | Agilent BioTek Gen 5 3.11.19, DRAGEN v3.10.12, BioRad ChromLab, Thermo Xcalibur, Bio-rad Image Lab 6.1, ThermoFisher QuantStudio5 |
| Data analysis | RAxML v8.2.12, OrthoFinder v2.5.5, MUSCLE v5.3, iTOL v6, breseq 0.37.1, trim-galore 0.6.7, featureCounts v2.0.1, DESeq2 1.44.0, Thermo FreeStyle, ProSite Lite, Excel 16.77.1, GraphPad Prism 10.5.0, and DESeq2 1.44.0. Code for obtaining data, running OrthoFinder and generating phylogenetic trees can be found at https://github.com/JonWinkelman/astR_trees_geary2025 with DOI 10.5281/zenodo.20397902. Code for RNA-seq analysis can be found at https://github.com/trestle-biosciences/rnaseq_prokaryotes with DOI 10.5281/zenodo.20612530. |

For manuscripts utilizing custom algorithms or software that are central to the research but not yet described in published literature, software must be made available to editors and reviewers. We strongly encourage code deposition in a community repository (e.g. GitHub). See the Nature Portfolio guidelines for submitting code & software for further information.

## Data

Policy information about availability of data

All manuscripts must include a data availability statement. This statement should provide the following information, where applicable:
- Accession codes, unique identifiers, or web links for publicly available datasets
- A description of any restrictions on data availability
- For clinical datasets or third party data, please ensure that the statement adheres to our policy

The sequencing data generated in this study have been deposited in the NCBI Gene Expression Omnibus and Sequence Read Archive databases under BioProject accession code PRJNA1328811. The mass spectrometry data generated in this study have been deposited in the MassIVE database under accession code

MSV000099253. All other source data have been deposited in the Zenodo database with DOI 10.5281/zenodo.20737328 and are provided as a Source Data file.

## Research involving human participants, their data, or biological material

Policy information about studies with human participants or human data. See also policy information about sex, gender (identity/presentation), and sexual orientation and race, ethnicity and racism.

| | |
|---|---|
| Reporting on sex and gender | The study does not include human participants or biological material. |
| Reporting on race, ethnicity, or other socially relevant groupings | The study does not include human participants or biological material. |
| Population characteristics | The study does not include human participants or biological material. |
| Recruitment | The study does not include human participants or biological material. |
| Ethics oversight | The study does not include human participants or biological material. |

Note that full information on the approval of the study protocol must also be provided in the manuscript.

# Field-specific reporting

Please select the one below that is the best fit for your research. If you are not sure, read the appropriate sections before making your selection.

☒ Life sciences ☐ Behavioural & social sciences ☐ Ecological, evolutionary & environmental sciences

For a reference copy of the document with all sections, see nature.com/documents/nr-reporting-summary-flat.pdf

# Life sciences study design

All studies must disclose on these points even when the disclosure is negative.

| | |
|---|---|
| Sample size | Sample size was determined based on previous research in the laboratory and norms in the field. For bacterial culture experiments, n=3 was used. For mouse experiments, n=6 (3 mice per cage) was used for 16S sequencing of uninoculated animals and n=5 was used for inoculated animals, which is typically sufficient power to detect significant differences in bacterial burdens in competitive inoculation experiments. |
| Data exclusions | No data were excluded from the analyses. |
| Replication | Experiments were replicated as indicated in the figure legend. Orogastric inoculation experiments were corroborated by competition experiments in fecal slurries from two independent cages of male and female mice. |
| Randomization | Samples were not randomized. For the mouse experiment, the covariates were controlled because every mouse received both strains. For mouse fecal slurry experiments, the covariates were controlled because each sample was tested with all bacterial strains. All other experiments were performed on bacterial cultures from randomly selected colonies. |
| Blinding | Blinding was not applied in this study as the experiments primarily involved objective measurements where outcomes were based on quantitative data rather than subjective interpretations, making blinding unnecessary. |

# Reporting for specific materials, systems and methods

We require information from authors about some types of materials, experimental systems and methods used in many studies. Here, indicate whether each material, system or method listed is relevant to your study. If you are not sure if a list item applies to your research, read the appropriate section before selecting a response.

## Materials & experimental systems

| n/a | Involved in the study | |
|---|---|---|
| ☐ | ☒ | Antibodies |
| ☒ | ☐ | Eukaryotic cell lines |
| ☒ | ☐ | Palaeontology and archaeology |
| ☐ | ☒ | Animals and other organisms |
| ☒ | ☐ | Clinical data |
| ☒ | ☐ | Dual use research of concern |
| ☒ | ☐ | Plants |

## Methods

| n/a | Involved in the study | |
|---|---|---|
| ☒ | ☐ | ChIP-seq |
| ☒ | ☐ | Flow cytometry |
| ☒ | ☐ | MRI-based neuroimaging |

## Antibodies

| | |
|---|---|
| Antibodies used | Invitrogen, Biotin-Anti-His-Tag Antibody, HIS.H8, MA1-21315, Lot ZA384901, dilution: 10/77 |
| Validation | The ThermoFisher Scientific website notes at least 328 citations using this antibody for Western blot (https://www.thermofisher.com/antibody/product/6x-His-Tag-Antibody-clone-HIS-H8-Monoclonal/MA1-21315) |

## Animals and other research organisms

Policy information about studies involving animals; ARRIVE guidelines recommended for reporting animal research, and Sex and Gender in Research

| | |
|---|---|
| Laboratory animals | Conventional mice were Swiss-Webster from Charles River and Envigo, male and female as indicated, 6 weeks old. Germ-free mice were C57BL/6J, male and female, 8-28 weeks old. Conventional fecal slurry experiments in Figure 4C used 6-week-old Swiss-Webster female mice from Charles River. Experiments in Figure 5 and S6A-B used 6-week-old Swiss-Webster female mice from Envigo. Conventional fecal slurry experiments in Figure S6C were 6-week-old Swiss-Webster male and female mice from Charles River. The fecal slurry experiment in Figure 4D used samples from female C57BL/6J mice approximately 10 weeks of age. |
| Wild animals | The study did not involve wild animals. |
| Reporting on sex | Female mice were used for in vivo studies because we previously reported no difference baesd on sex for a similar phenotype (Ren and Clark et al., Cell Host & Microbe, 2025). Fecal samples from male and female mice were homogenized to test bacterial reporter activity and displayed similar results. Fecal samples from male and female mice were homogenized to test bacterial competition ex vivo and male and female mice are indicated by symbol shape, as reported in the supplementary figure and Source Data. |
| Field-collected samples | The study did not involve samples collected from the field. |
| Ethics oversight | Animal care protocols were approved by the University of Illinois Chicago (UIC) Institutional Animal Care and Use Committee (protocol numbers 23-119 and 22–192) according to the Animal Care Policies at UIC, the Animal Welfare Act, the National Institute of Health and the American Veterinary Medical Association. |

Note that full information on the approval of the study protocol must also be provided in the manuscript.

## Plants

| | |
|---|---|
| Seed stocks | The study did not use plant material. |
| Novel plant genotypes | The study did not produce novel plant genotypes. |
| Authentication | The study did not produce seed stock or novel plant genotype. |
