# Supplementary material for: A regulator of amino acid catabolism controls Acinetobacter baumannii gut colonization
Source: Nat Commun. 2026 Aug 14;17:9773. doi: 10.1038/s41467-026-75861-5 (PMC13572408; doi:10.1038/s41467-026-75861-5)
Supplement: Supplementary file 4 [file 41467_2026_75861_MOESM8_ESM.pdf]

nature portfolio

Peer Review File

A regulator of amino acid catabolism controls *Acinetobacter baumannii* gut colonization

Corresponding Author: Professor Lauren Palmer

Parts of this Peer Review File have been redacted as indicated to maintain the confidentiality of unpublished data.

This file contains all reviewer reports in order by version, followed by all author rebuttals in order by version.

Version 0:

Reviewer comments:

Reviewer #1

(Remarks to the Author)
Acinetobacter gut colonization increases the risk for subsequent infection. Prior work by this team revealed that ornithine utilization is critical for A. baumannii (Abau) competitive fitness in the gut. This manuscript expands the prior work by examining the regulatory mechanisms controlling ornithine utilization by Abau. This study identified AstR divergently transcribed from astNOP and proposes that AstR was co-opted from arginine utilization to specifically regulate ornithine utilization (astNOP) in Abau. The astN promoter is activated by ornithine, but inhibited by glutamate and other preferred amino acids. The astN promoter is also activated in fecal samples from conventional mice, not germ-free mice and AstR is required Abau gut colonization. Overall, the manuscript is clear and the conclusions are mostly supported by the current data. The few major suggestions are aimed to strengthen the alignment of data and conclusions.

Major
1. All conclusions are drawn from reporter plasmids and RNASeq. Key results ought to be validated with qRT-PCR on astR, astN, astO, and astP transcripts.
2. The negative MS and SEC results do not necessarily mean that AstR does not have a ligand as concluded. Is it possible that the AstR ligand is labile? Would cross-linking prior to MS or SEC change the spectral signatures?
3. Does ornithine, arginine, glutamate or other suppressive amino acids differ between conventional and GF fecal slurries. There are commercial kits that measure individual amino acids and ornithine. If orn/arg is higher in conventional feces, or suppressive amino acids are higher in GF that would greatly strengthen the proposed model and impact.

Minor
1. Line 40: specify that this is in mice
2. Line 79-81: Authors claim AstR is divergently transcribed from astNOP in Abau complex pathogens, but data supporting conservation of astR/astNOP is not provided. How frequently are the loci presented in 1B present in A. baumannii, colistiniresistens, or baylyi genomes? Also the text lacks clarity due to missing details about Acinetobacter phylogeny that non-experts have not yet been introduced to. Perhaps revise to: "…only in the Abau/calc complex, which contains all…"
3. Line 82-85: It is unclear if there is a regulator associated with the primary A bau ast locus. How similar is it to astR?
4. Fig 1D: It is hard to distinguish the arms of the phylogeny. Is it possible to extend the bracket arms identifying Acinetobacter related-ness for clarity?
5. Line 134-136 – From Fig S2B, it appears that the astR promoter may be constitutively active, and not upregulated by the presence of arginine/ornithine. However, Fig 2A suggests that activation of the astNOP promoter is upregulated in upon addition of arginine/ornithine in an AstR-dependent manner. It might be interesting to expand speculations on the regulation occurring here. Fig 3 shows that AstR binds to the astNp promoter in a concentration-dependent manner, and it is possible that another factor may bind at the -100 to -135 site you found important for AstR binding to occlude it until Arginine/ornithine is detected.
6. Fig S2C – the figure legend does not describe left sub-panel. Is this astNp a positive control?
7. Fig 2A/S2A – It would be informative to add the RNASeq conditions (orn/succ – no NH4) to directly compare astNp-lux and RNA-Seq results, as in S2D.
8. Lines 139-141 – Figure 2C indicates that ΔastR was grown in succinate/ornithine to assess gene expression changes by RNAseq compared to WT, but Fig 1E indicates that under these conditions, ΔastR does not grow. Although the authors

specified the succ/orn was for 1 hr, perhaps add a few extra words emphasizing that a temporary exposure was used due to astR viability.
9. Fig S3D-F: it is unclear why succ/arg combination was tried, but not orn/succ. Data support that the highest astNp activation occurs in orn/succ.
10. Line 262 – is succinate a typo? Do you mean ornithine?

(Remarks on code availability)

Reviewer #2

(Remarks to the Author)
This manuscript by Geary & Ren et al. reports the findings of a study aimed at interrogating the role of the Acinetobacter baumannii (Ab) transcriptional regulator AstR. This regulator appears to have diverged from other paralogs to transcriptionally control the orphaned astNOP operon for ornithine utilization. The strengths of the manuscript include:

1. Clear, concise, and effective presentation.
2. Thorough complementary data that strengthen the authors' conclusions (Figs. 1E and 1F & S1B and S1C).
3. Appropriate statistics are used throughout the manuscript.
4. Fulfillment of the molecular Koch's postulates to demonstrate the necessity of AstR.
5. The molecular interrogation of Ab colonization, an explicit risk factor for infection, is traditionally a challenging pathogen to study and, thus, less well-characterized compared to other AMR pathogens.

Overall, this is an exciting study; however, there are some concerns regarding the confidence in the interpretation of the study data that are outlined below:

Major concerns

1. More confirmatory data demonstrating gut microbial community recovery following gentamicin treatment should be presented for Figure 5, as in Figure 3 of the authors' prior study (reference 18). Without these data, these experiments lack the conclusive context that ties AstR to AstO via ornithine, leading to colonization success.

Minor concerns
1. It would be helpful if the introduction included a more thorough review of ornithine metabolism for those not familiar with these metabolic pathways.

2. It would be compelling to repeat the experiments in Figs. 1E and 1F with A. colistiniresistens to understand if the role of AstR regulation of AstO is specific to the Ab complex or more broadly applicable, given that their orphaned operon differs (Fig. 1B). Such an experiment would strengthen and contextualize the authors' conclusions.

3. The logic for using succinate in Fig. 2A is sound; however, Figs. 2A and S2A, panel 6 (bottom row, second from left) indicates that AstR is non-responsive to Orn/NH4 in the WT background (e.g. no astN transcription). This is seemingly discordant with these data in Fig. 2B (e.g. lots of astN transcript). Presumably, there is some level of astN transcription in the WT background in LB, but no data supporting this presumption is shown. How do the authors explain these data?

4. Please clarify if the experiments in Fig. 4C were performed aerobically or anaerobically.

5. Related to Major concern 1, it may be helpful to use ornithine supplementation to confirm the insensitivity of the AstR strain to exogenous ornithine in a whole animal system, as in Figure 3 of the authors' prior study (reference 18).

6. Are there any clinical or observation data that validate the role of ornithine in the context of Ab colonization? For example, are individuals on a ketogenic diet (high ornithine levels) more susceptible to Ab colonization?

(Remarks on code availability)
READMEs are available, and installation and application are clear.

Reviewer #3

(Remarks to the Author)
I co-reviewed this manuscript with one of the reviewers who provided the listed reports. This is part of the Nature Communications initiative to facilitate training in peer review and to provide appropriate recognition for Early Career Researchers who co-review manuscripts.

(Remarks on code availability)

Version 1:

Reviewer comments:

Reviewer #1

(Remarks to the Author)
I have carefully reviewed the revised manuscript. The authors thoughtfully and thoroughly responded to my prior concerns.

Minor: There appears to be a word missing [related] in the new line 98: "complex is more closely [related] to the occasionally…"

Otherwise, I have no further concerns.

(Remarks on code availability)

Reviewer #2

(Remarks to the Author)
Thank you for addressing my concerns. The authors should be proud of this excellent work. My only remaining comment is that WTp-empty and astRp-empty in the legend of Figure 1F-G are incorrect (the legend symbols don't match the graph symbols).

(Remarks on code availability)
READMEs are available, and installation and application are clear.

Reviewer #3

(Remarks to the Author)
I co-reviewed this manuscript with one of the reviewers who provided the listed reports. This is part of the Nature Communications initiative to facilitate training in peer review and to provide appropriate recognition for Early Career Researchers who co-review manuscripts.

(Remarks on code availability)

Open Access This Peer Review File is licensed under a Creative Commons Attribution 4.0 International License, which permits use, sharing, adaptation, distribution and reproduction in any medium or format, as long as you give appropriate credit to the original author(s) and the source, provide a link to the Creative Commons license, and indicate if changes were made.
In cases where reviewers are anonymous, credit should be given to 'Anonymous Referee' and the source.
The images or other third party material in this Peer Review File are included in the article’s Creative Commons license,

unless indicated otherwise in a credit line to the material. If material is not included in the article's Creative Commons license and your intended use is not permitted by statutory regulation or exceeds the permitted use, you will need to obtain permission directly from the copyright holder.
To view a copy of this license, visit https://creativecommons.org/licenses/by/4.0/

Summary of response:

We thank the Reviewers for their thoughtful comments on our manuscript. In this document, we provide a point-by-point response to Reviewer comments and describe substantial new data we added to the manuscript to address these points. Specifically, we present new data demonstrating that the occasionally pathogenic *Acinetobacter* species *A. colistiniresistens* AstR complement growth for *A. baumannii* ∆*astR* and *A. baylyi* ∆*astR* (new Figure 1F-G). Although *A. colistiniresistens* is more closely related to *A. baumannii* (new Figure 1D), these data suggest that *A. colistiniresistens* AstR is more functionally similar to *A. baylyi* AstR, as suggested by the phylogenetic analysis in Figure 1E. We further explored the prevalence of AstR and the *ast* loci among *Acinetobacter* species and found substantial variability (new Figure S1B, new Table S1). We additionally investigated whether supplementation of ornithine altered competition of wildtype (WT) and ∆*astR* in the gut and whether it changed the gut microbiota. We provide new data that dietary supplementation with ornithine promotes WT but not ∆*astR* colonization in gut (new Figure 5A-C, S6A). However, the gut microbiota was not dramatically changed (new Figure 5D-E, S6B). This suggests that dietary ornithine supplementation directly promotes *A. baumannii* colonization in the gut through AstR-regulated ornithine catabolism, rather than indirect effects through the microbiota. Together, the clarifications and new experiments strengthen the rigor and conclusions of the manuscript.

Line numbers refer to the marked version of the manuscript.

Reviewer #1 (Remarks to the Author):
Acinetobacter gut colonization increases the risk for subsequent infection. Prior work by this team revealed that ornithine utilization is critical for A. baumannii (Abau) competitive fitness in the gut. This manuscript expands the prior work by examining the regulatory mechanisms controlling ornithine utilization by Abau. This study identified AstR divergently transcribed from astNOP and proposes that AstR was co-opted from arginine utilization to specifically regulate ornithine utilization (astNOP) in Abau. The astN promoter is activated by ornithine, but inhibited by glutamate and other preferred amino acids. The astN promoter is also activated in fecal samples from conventional mice, not germ-free mice and AstR is required Abau gut colonization. Overall, the manuscript is clear and the conclusions are mostly supported by the current data. The few major suggestions are aimed to strengthen the alignment of data and conclusions.

Major

1. All conclusions are drawn from reporter plasmids and RNASeq. Key results ought to be validated with qRT-PCR on astR, astN, astO, and astP transcripts.

Response 1: The qRT-PCR data was added (new Figure S2C). We validated the transcript level changes of *astN*, *astO*, *astP*, and *ACX60_10810* gene in minimal medium with ornithine as the sole carbon or nitrogen source. The *astN* and *astO* transcripts were significantly lower in ∆*astR* compared to WT in M9 ornithine/NH_4 and all transcripts were significantly lower in ∆*astR* compared to WT in M9 succinate/NH_4.

2. The negative MS and SEC results do not necessarily mean that AstR does not have a ligand as concluded. Is it possible that the AstR ligand is labile? Would cross-linking prior to MS or SEC change the spectral signatures?

Response 2: Thank you for your recommendation. We tried multiple times to detect potential ligands after co-incubating with purified His_6-AstR by native MS. However, due

to technical issues these experiments were not successful. We revised the manuscript to clarify this point (Line 328-330, Figure S4 title).

3. Does ornithine, arginine, glutamate or other suppressive amino acids differ between conventional and GF fecal slurries. There are commercial kits that measure individual amino acids and ornithine. If orn/arg is higher in conventional feces, or suppressive amino acids are higher in GF that would greatly strengthen the proposed model and impact.

Response 3: Thank you for this question. We previously published amino acid metabolome data in the feces of conventional mice and germ-free mice (Fig. 3E and Fig. S4I, Ren and Clark, 2025 *Cell Host & Microbe*). Here, we show the ornithine, arginine, glutamate, and histidine concentrations on day 0 in conventional mice (with or without gentamicin antibiotics (Abx) pretreatment) and germ-free mice. The levels of ornithine and glutamate was lower in the germ-free mice compared to Abx-treated conventional mice. The levels of arginine, histidine and asparagine were higher in the germ-free mice compared to Abx-treated conventional mice. In our previous data, glutamate, histidine, and asparagine were preferred carbon sources over arginine and ornithine. The lower expression of *astNp* in GF mice fecal slurry might be due to the high abundance of preferred carbon sources such as histidine and asparagine. We added text addressing these previously reported data (Lines 317-319).

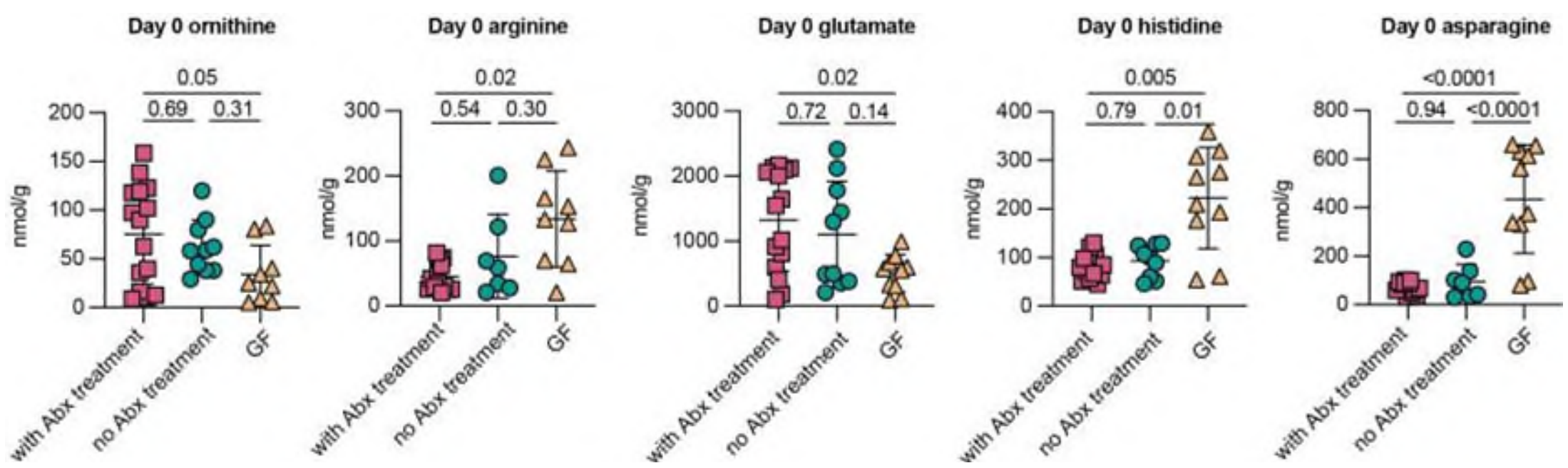

Minor

4. Line 40: specify that this is in mice

Response 4: These data were from human infants, which we clarified in the text (Line 40).

5. Line 79-81: Authors claim AstR is divergently transcribed from astNOP in Abau complex pathogens, but data supporting conservation of astR/astNOP is not provided. How frequently are the loci presented in 1B present in A. baumannii, colistiniresistens, or baylyi genomes? Also the text lacks clarity due to missing details about Acinetobacter phylogeny that non-experts have not yet been introduced to. Perhaps revise to: "...only in the Abau/calc complex, which contains all..."

Response 5. Thank you. We clarified this point as recommended. We previously reported that that the complete *ast2* locus (*astR, astNOP*) was present in 83.9% of *A. baumannii* isolates, that a partial *ast2* locus was present in 8.5% of *A. baumannii* isolates, and that there was no *ast2* locus in 7.6% of *A. baumannii* (Ren and Clark *et al.*, *Cell Host & Microbe*, 2025). This suggested that the *ast2* locus is conserved in the majority of sequenced *A. baumannii* isolates.

For the conservation of *astR*, *ast(G)CADBE*, and *astNOP* among *Acinetobacter* species, we explored 221 representative *Acinetobacter* species genomes. In these representative

genomes, 41.2% of *Acinetobacter* genomes encode *astR* and at least one *ast* locus. Specifically, 14% *Acinetobacter* genomes encode *astR* and the complete *astNOP* locus(as depicted for the *A. baumannii* complex in Figure 1B); 10.9% of *Acinetobact*er genomes encode *astR* with a partial *ast2* locus (as depicted for the *A. colistiniresistens* complex in Figure 1B), 16.3% of *Acinetobacter* isolates only encode *astR* with *ast(G)CADBE* locus and lack the *astNOP* locus (as depicted for the *A. baylyi* complex in Figure 1B) (Lines 108-118). We depict these findings in new Figure S1B and new Table S1.

6. Line 82-85: It is unclear if there is a regulator associated with the primary A bau ast locus. How similar is it to astR?

Response 6. There is no regulator associated with the *astGCADBE* operon in *A. baumannii*. As mentioned in the manuscript, another group reported that GacA regulates the *ast*GCADBE operon in *A. baumannii* by direct or indirect mechanisms (Lines 333-335) (Kuldip *et al*., *Infect Immun*, 2025). **REDACTED: CONFIRMED BY HANDLING EDITOR We clarified that there is no regulator encoded upstream of the *astGCADBE* operon in *A. baumannii* in the text (Lines 337-338).**

7. Fig 1D: It is hard to distinguish the arms of the phylogeny. Is it possible to extend the bracket arms identifying Acinetobacter related-ness for clarity?

Response 7. Thank you for this suggestion. We edited the AstR protein tree (now Figure 1E) and removed the outgroup for clarity.

8. Line 134-136 – From Fig S2B, it appears that the astR promoter may be constitutively active, and not upregulated by the presence of arginine/ornithine. However, Fig 2A suggests that activation of the astNOP promoter is upregulated in upon addition of arginine/ornithine in an AstR-dependent manner. It might be interesting to expand speculations on the regulation occurring here. Fig 3 shows that AstR binds to the astNp promoter in a concentration-dependent manner, and it is possible that another factor may bind at the -100 to -135 site you found important for AstR binding to occlude it until Arginine/ornithine is detected.

Response 8. Thank you for this thoughtful comment. **REDACTED: CONFIRMED BY HANDLING EDITOR We added additional text discussing these possibilities (Lines 332-333).**

9. Fig S2C – the figure legend does not describe left sub-panel. Is this astNp a positive control?

Response 9. Yes, the *astNp* was a positive control. We edited the figure legend (now Figure S2D).

10. Fig 2A/S2A – It would be informative to add the RNASeq conditions (orn/succ – no NH4) to directly compare astNp-lux and RNA-Seq results, as in S2D.

Response 10. We have added the *astNp*-lux expression data in succ/orn medium without NH_4 in Figure 2A and Figure S2A. We clarified that in the text (Lines 148-149).

11. Lines 139-141 – Figure 2C indicates that ΔastR was grown in succinate/ornithine to assess gene expression changes by RNAseq compared to WT, but Fig 1E indicates that under these conditions, ΔastR does not grow. Although the authors specified the succ/orn was for 1 hr, perhaps add a few extra words emphasizing that a temporary exposure was used due to astR viability.
Response 11. Thank you. We edited the main text to clarify this point (Lines 161-163).

12. Fig S3D-F: it is unclear why succ/arg combination was tried, but not orn/succ. Data support that the highest astNp activation occurs in orn/succ.

Response 12. We added new EMSA data where the AstR protein was incubated with ornithine and succinate (<u>new Figure S3G</u>). This assay showed that the combination ornithine and succinate also did not dramatically alter His_6-AstR binding to *astNp* DNA by EMSA.

13. Line 262 – is succinate a typo? Do you mean ornithine?

Response 13. Thank you. This was not a typo. We have edited the main text to clarify "Data included here show AstR-dependent activation of the *astNOP* operon requires arginine/ornithine and is enhanced in the presence of the organic acid succinate"(Lines 308-310).

Reviewer #2 (Remarks to the Author):
This manuscript by Geary & Ren et al. reports the findings of a study aimed at interrogating the role of the Acinetobacter baumannii (Ab) transcriptional regulator AstR. This regulator appears to have diverged from other paralogs to transcriptionally control the orphaned astNOP operon for ornithine utilization. The strengths of the manuscript include:

1. Clear, concise, and effective presentation.
2. Thorough complementary data that strengthen the authors' conclusions (Figs. 1E and 1F & S1B and S1C).
3. Appropriate statistics are used throughout the manuscript.
4. Fulfillment of the molecular Koch's postulates to demonstrate the necessity of AstR.
5. The molecular interrogation of Ab colonization, an explicit risk factor for infection, is traditionally a challenging pathogen to study and, thus, less well-characterized compared to other AMR pathogens.

Overall, this is an exciting study; however, there are some concerns regarding the confidence in the interpretation of the study data that are outlined below:

Major concerns

14. More confirmatory data demonstrating gut microbial community recovery following gentamicin treatment should be presented for Figure 5, as in Figure 3 of the authors' prior study (reference 18). Without these data, these experiments lack the conclusive context that ties AstR to AstO via ornithine, leading to colonization success.

Response 14. Thank you for this suggestion. We performed the mouse experiment after gentamicin treatment and with or without 1% ornithine supplementation (<u>new Figure 5A</u>). From this experiment, we performed 16S rRNA gene sequencing from day -11 (before

gentamicin), day 0, and day 9 feces. Ornithine supplementation promoted the WT colonization but did not rescue Δ*astR*, similarly to as we previously observed for Δ*astO* (Ren and Clark *et al.*, 2025; new Figure 5B-C*).* Additionally, the gut microbiota recovered to same alpha-diversity by day 9 compared to day -11 before antibiotics (new Figure 5D) and more similar beta-diversity and amplicon sequence variant (ASV) profiles compared to day 0 (Figure 5E, S6B). The ornithine supplementation did not dramatically change the microbiota compared to no supplementation (new Figure 5D-E, S6B). This suggests that the dietary ornithine directly promotes *A. baumannii* AstR-dependent ornithine catabolism for gut colonization.

Minor concerns

15. It would be helpful if the introduction included a more thorough review of ornithine metabolism for those not familiar with these metabolic pathways.

Response 15. Thank you for this point. We added a brief discussion of the sources of ornithine in the gut (Lines 49-50).

16. It would be compelling to repeat the experiments in Figs. 1E and 1F with *A. colistiniresistens* to understand if the role of AstR regulation of AstO is specific to the Ab complex or more broadly applicable, given that their orphaned operon differs (Fig. 1B). Such an experiment would strengthen and contextualize the authors' conclusions.

Response 16. Thank you for this suggestion. We made new constructs and strains where *A. baumannii* Δ*astR* and *A. baylyi* Δ*astR* were complemented by *astR*$_{A.\ colistiniresistens}$ and repeated these experiments. The *astR*$_{A.\ colistiniresistens}$ complemented the growth for *A. baumannii* Δ*astR* and *A. baylyi* Δ*astR* (new Figure 1F-G). This suggests that the AstR from *A. colistiniresistens* has similar function as AstR from *A. bayly*i, consistent with the phylogenetic tree showing that *A. colistiniresistens* AstR is more closely related to *A. baylyi* AstR (Figure 1E). We added text describing these results (Lines 136-140).

17. The logic for using succinate in Fig. 2A is sound; however, Figs. 2A and S2A, panel 6 (bottom row, second from left) indicates that AstR is non-responsive to Orn/NH4 in the WT

background (e.g. no astN transcription). This is seemingly discordant with these data in Fig. 2B (e.g. lots of astN transcript). Presumably, there is some level of astN transcription in the WT background in LB, but no data supporting this presumption is shown. How do the authors explain these data?

Response 17. Thank you for this point. *astN* was expressed in all wild-type conditions assessed by RNA-seq: expression was very low in succ/NH_4, then slightly induced in Orn/NH_4, then induced more dramatically in succ/Orn. To address this point, we added a volcano plot of the RNA-seq data depicting fold-changes of expression in WT comparing (Orn/NH_4)/(succinate/NH_4) (<u>new Figure S2E left panel</u>). These data show that for WT *A. baumannii*, *astN* and *astO* are upregulated in Orn/NH_4 compared to Succ/NH_4. However, as shown by fold-changes for *ast* genes in WT (succinate/Orn)/(Orn/NH_4) already included in Figure S2E (now Figure S2E right panel), the magnitude of *astNO* activation in Orn/NH_4 is much lower compared to succinate/Orn.

We have not measured the *ast* transcript abundance in LB medium; however, a published RNA-seq dataset shows that *astR* is expressed in LB and *astN* is expressed with many transcript reads in the 5' end of the gene with a sharp drop off (Kroger *et al.*, *Nucl. Acids Res.*, 2018), suggesting there may be post-transcriptional regulation. **REDACTED: CONFIRMED BY HANDLING EDITOR**

18. Please clarify if the experiments in Fig. 4C were performed aerobically or anaerobically.

Response 18: We edited the text to clarify, "In fecal slurries from conventional mice incubated aerobically" (Line 249).

19. Related to Major concern 1, it may be helpful to use ornithine supplementation to confirm the insensitivity of the AstR strain to exogenous ornithine in a whole animal system, as in Figure 3 of the authors' prior study (reference 18).

Response 19: Thank you for this suggestion. We performed the 1% ornithine supplementation in conventional mice experiment. Our data showed that ornithine

supplementation promotes gut colonization WT but not z*astR A. baumannii* (new Figure 5B-C, S6A). These results confirmed that the z*astR* strain is insensitive to exogenous ornithine to promote *A. baumannii* gut colonization in a physiologically relevant environment.

20. Are there any clinical or observation data that validate the role of ornithine in the context of Ab colonization? For example, are individuals on a ketogenic diet (high ornithine levels) more susceptible to Ab colonization?

Response 20: Thank you for this suggestion. Since *A. baumannii* colonization is typically low abundance, unfortunately there are few studies reporting *Acinetobacter* abundance when comparing diet. We added discussion of some studies showing association of diet with *Acinetobacter* spp. or *A. baumannii* gut colonization (Lines 296-299).

Reviewer #2 (Remarks on code availability):
READMEs are available, and installation and application are clear.

<u>Summary of response:</u>
We thank the Reviewers for their careful review and positive comments. We provide the additional requested revisions.

<u>Reviewer #1 (Remarks to the Author):</u>
I have carefully reviewed the revised manuscript. The authors thoughtfully and thoroughly responded to my prior concerns.

Comment 1: Minor: There appears to be a word missing [related] in the new line 98: "complex is more closely [related] to the occasionally…"
Otherwise, I have no further concerns.

Response 1: Thank you for noticing this omission, which we have corrected.

<u>Reviewer #2 (Remarks to the Author):</u>
Thank you for addressing my concerns. The authors should be proud of this excellent work.

Comment 2: My only remaining comment is that WTp-empty and astRp-empty in the legend of Figure 1F-G are incorrect (the legend symbols don't match the graph symbols).

Response 2. Thank you for noticing this mistake, which we have corrected.
